# Diagnosis, Evaluation, and Management of Wildlife, Exotic and Zoo Animals’ Diseases—Advances and Challenges

**DOI:** 10.3390/ani16081211

**Published:** 2026-04-16

**Authors:** Isabel Pires, Andreia Garcês, Filipe Silva

**Affiliations:** 1 CECAV—Veterinary and Animal Research Center, University of Trás-os-Montes e Alto Douro, 5000-801 Vila Real, Portugal; andreiagarces@utad.pt (A.G.); fsilva@utad.pt (F.S.); 2 Veterinary Sciences Department, University of Trás-os-Montes e Alto Douro, 5000-801 Vila Real, Portugal

It is a privilege to serve as Guest Editor for this Topical Collection of *Animals* dedicated to wildlife, exotic, and zoo animal health and disease. The contributions in this issue reflect the complexity of disease processes across diverse species and the need to understand them within an evolving biological, ecological, and environmental context. The editorial team behind this Topical Collection brings together both pathologists and clinicians, combining complementary expertise in wildlife and exotic animal medicine. In addition to their work on infectious, parasitic, and metabolic diseases, the editors have developed a growing interest in neoplasia in these animals. This line of research is particularly relevant, as tumors in wildlife and zoo species may provide important insights into how environmental factors influence cancer development within a broader One Health framework [1].

Disease in wildlife and zoo animals cannot be interpreted through a static lens. Instead, it must be approached evolutionarily, in which dynamic interactions among hosts, pathogens, and changing environments shape etiology, pathogenesis, and structural and functional changes. Increasing anthropogenic pressure, habitat fragmentation, climate change, and intensified human–animal interfaces further complicate these interactions, reinforcing the importance of integrative approaches to animal health [2,3]. One central concept highlighted throughout this collection is the role of wildlife as sentinels of ecosystem health. Several articles emphasize how wild animals can act as reservoirs of infectious agents, indicators of environmental contamination, and early warning systems for emerging diseases. Studies addressing zoonotic and bacterial pathogens—such as *Salmonella*, *Coxiella burnetii*, antimicrobial-resistant *Enterobacteria*, and *Erysipelothrix rhusiopathiae* demonstrate the relevance of wildlife in complex epidemiological cycles with implications for both animal and human health.

The detection of viral agents at the human–animal interface, including reports of SARS-CoV-2 in non-human primates and other wildlife species, further reinforces the importance of surveillance systems that integrate veterinary, ecological, and public health perspectives. These findings highlight the bidirectional nature of disease transmission and the need for a One Health approach.

This Topical Collection also showcases the importance of retrospective and population-based analyses. Long-term datasets, such as the multi-institutional review of platypus medical records, as well as studies from wildlife rehabilitation centers, provide valuable insights into disease patterns, causes of admission, clinical outcomes, and opportunities to improve treatment strategies and conservation efforts. Such approaches are essential for species that are difficult to study in the wild or for which baseline health data remain limited.

In addition, several contributions focus on diagnostics and clinical reference data, which are fundamental for advancing veterinary care in non-traditional species. Studies establishing biochemical and physiological reference values, as well as those employing advanced imaging techniques, contribute to more accurate diagnosis and improved clinical decision-making in both captive and free-ranging animals.

The influence of management conditions and the environment on animal health is another key theme. Differences in gut microbiota between captive and semi-free-ranging animals, as well as disease patterns associated with captivity, underline how husbandry, nutrition, and environmental exposure shape health outcomes. These findings are particularly relevant to zoos and conservation programs that aim to balance animal welfare with species preservation.

Parasitological, microbiological, and pathological investigations included in this collection further expand our understanding of biodiversity and host–pathogen interactions. At the same time, applied studies, such as those evaluating disease-detection tools or assessing risks associated with wildlife translocation, demonstrate how scientific knowledge can be translated into practical solutions for conservation and disease management.

A total of twenty-one articles were published in this Topical Collection, encompassing original research and reviews across a wide range of species, regions, and disciplines. Together, they illustrate the multidisciplinary nature of wildlife medicine and the importance of integrating clinical practice, diagnostics, epidemiology, and conservation biology.

Despite these advances, significant knowledge gaps remain. For many species, basic clinical parameters, disease descriptions, and epidemiological data are still scarce. Addressing these gaps requires continued collaboration across institutions and disciplines, as well as the systematic collection and sharing of data from field studies, zoological institutions, and rehabilitation centers.

In conclusion, this Topical Collection underscores the need for a holistic, integrative perspective on disease in wildlife, exotic, and zoo animals. Understanding animal health in this context is essential not only for improving individual welfare but also for supporting conservation strategies, safeguarding ecosystem health, and mitigating risks to human populations.

We would like to sincerely thank all authors for their valuable contributions and the reviewers for their expertise and dedication. I also extend my gratitude to the editorial team of *Animals* for their continuous support, especially to Ms. Vicky Liu and Mrs. Ivy Zhang. We hope this collection will advance knowledge, foster collaboration, and guide future research in wildlife and conservation medicine.
